# Characteristics of Dissolved Organic Nitrogen in the Sediments of Six Water Sources in Taihu Lake, China

**DOI:** 10.3390/ijerph16060929

**Published:** 2019-03-14

**Authors:** Xiaofan Yang, Xueyu Wei, Xiaoping Xu, Yu Zhang, Jincheng Li, Jie Wan

**Affiliations:** College of Biological and Chemical Engineering, Anhui Polytechnic University, Wuhu 24100, China; xiaofan108@ahpu.edu.cn (X.Y.); xuxp1979@126.com (X.X.); nbz022@126.com (Y.Z.); wjxfy108@126.com (J.L.); valleyvan.1983@aliyun.com (J.W.)

**Keywords:** Taihu Lake, water source, sediment, dissolved organic nitrogen

## Abstract

KCl-extractable sediment dissolved organic nitrogen (KS-DON) extracted from sediments near drinking water intakes of six drinking water sources in Taihu Lake in China was partitioned into hydrophobic and hydrophilic fractions and high/low molecular weight fractions. The results showed that the total dissolved nitrogen (TDN) contents of the extracts ranged from 67.78 to 128.27 mg/kg. KS-DON was the main TDN species, accounting for more than 50%, with NH_4_^+^-N and NO_3_^−^-N averaging 30% and 20%, respectively. The molecular weight fractions of <1 kDa accounted for almost half of KS-DON. Hydrophilic compounds accounted for more than 75% of KS-DON. Three fluorescence peaks were identified: soluble microbial byproducts (A); protein-like substances (B); and humic acid-like substances (C). It is concluded that the KS-DON in Taihu Lake sources has higher bioavailability and higher risk of endogenous release. Ecological dredging and establishment of constructed wetlands are possible measures to reduce the release of endogenous nitrogen.

## 1. Introduction

Nitrogen is an essential element to living organisms, particularly in primary productivity in aquatic ecosystems [1]. Dissolved organic nitrogen (DON) is the important and active component of dissolved organic matter (DOM) in lake sediments, and approximately 12–72% of DON can be used as a nitrogen source by bacteria and algae [2]; thus, its availability and mobility have an important role in the dynamic processes of nitrogen mineralization, immobilization, leaching, and plant absorption, which are all important in nitrogen cycling in lake ecosystems [3]. The DON pool comprises a mix of complex compounds. Urea, dissolved free amino acids, proteins, nucleic acids, amino sugars, and humic substances have frequently been observed [4]. Kemp and Mudrochova [5] studied the surface sediments of Lake Ontario in Canada and reported that DON was the main component of TDN in surface sediments, accounting for 90% of the total nitrogen content of the sediment: 28–46% as amino acid nitrogen, 4–7% as hexosamine nitrogen, and 21–31% as hydrolyzable unidentified nitrogen. Lin et al. [6] researched six lakes, including Poyang Lake, and Dongting Lake in Hunan and Jiangxi Provinces, China, and reported that the DON content varied from 17.18 mg/kg to 292.31 mg/kg (mean content, 134.45 mg/kg), comprising 51.86% TDN and 7.14% total nitrogen (TN) across the six lakes; in addition, the distribution of DON was closely related to pollution levels in each lake. The sources of DON are extensive, including sediment, organic matter in the soil, microbial and biological organic debris, and their metabolites [7], besides, the effluent discharged from wastewater treatment plants is also an important source of anthropogenic DON loading to surface waters [8,9], with extremely complex composition [10] that can be bioavailable to aquatic plant species and toxic risk [11].

Many studies have confirmed the bioavailability of DON in sediments [12,13,14], indicating that higher content and fluorescence characteristics of hydrophilic fraction of dissolved organic matter (HIM) had a higher bioavailability and might remarkably impact the nitrogen cycle in water [15], and benthic macroalgae can also use low molecular weight DON compounds in sediments for growth. Besides, sediment DON can easily be released into overlying water in high temperature (>25 °C) and absorbed by algae quickly. Several research has used first-order kinetic equation to simulate the release kinetics of DON in the surface sediment [16,17]. Shi et al. [17] reported that the release kinetics of DON in the surface sediment could be well simulated by first-order kinetics equation, ranging from 24.387 to 46.949 mg/kg. Under certain conditions, the release flux of DON in shallow lake sediments is much greater than that of inorganic nitrogen [18].

Although DON content is usually low in surface water, it has become a critical issue in terms of drinking water treatment, because of reactions with chlorine that generate disinfection byproducts (DBPs) during the disinfection/oxidation processes [19,20]. Chlorine was found to react with DON released from sediment in a drinking water source during the disinfection process, resulting in the formation of nitrogenous (N)-DBPs [19,20], such as nitrosamines, halonitromethanes, and haloacetonitriles. Toxicity results indicated that N-DBPs are more strongly carcinogenic or mutagenic than are regulated DBPs, such as THMs and HAAs [21,22,23]. Thus, emerging concerns for drinking water safety have increased the need to understand better the concentration and speciation characteristics of DON.

Taihu Lake is an important source of drinking water in eastern China, with an important role in regional economic and social development, in addition to impacting human quality of life and wellbeing [24,25]. The northern and northwestern regions of Taihu Lake are the main inflow areas of the Taihu Lake Basin [26]. Every year, large amounts of TN and ammonia are discharged into Taihu Lake from the inflow rivers [27]. This high nitrogen input leads to an increase in nitrogen content in the western and northwestern regions of Taihu Lake [28] and, as a result of hydrodynamics and biological transformation processes, nitrogen is gradually deposited in the sediment [29,30], resulting in elevated concentrations of TDN and NH4+-N in sediments sampled in the western and northwestern regions of Taihu Lake compared with other regions. In addition, sediment nitrogen release [31] and extreme weather events (heavy rainfall and strong winds) [32] has been confirmed to be remarkably correlative with cyanobacterial bloom, threatening the water quality security of drinking water sources of Taihu Lake. In this study, the fluorescence, hydrophilicity/hydrophobicity and molecular weight distribution of DON in sediments of Lake Taihu were studied, which provided a theoretical basis for protection of Taihu Lake drinking water sources in the future.

## 2. Material and Methods

### 2.1. Study Site

**Figure 1:**
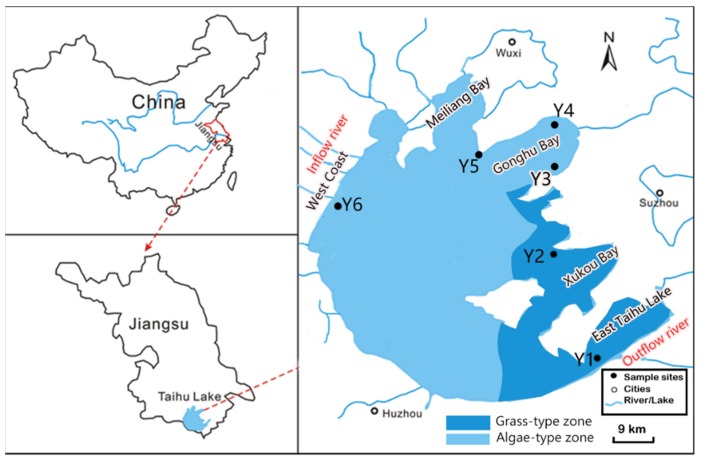
Sampling points in Taihu Lake.

Taihu Lake (119°52′ E–120°36′ E, 30°55′ N–31°32′ N), with an area of 2425 km^2^ and a mean depth of 1.90 m, is the third largest shallow freshwater lake in China, located in the southern part of the Yangtze River Delta, China [33] (Figure 1). Taihu Lake can be divided into an algae-type zone and a grass-type zone (Figure 1). In recent years, the boundary of algae-type zone and grass-type zone has been determined through field observations of the spatial distribution of aquatic vegetation and algae occurrence. Algal blooms outbreak frequently in the algae-type zone [34]. In the grass-type zone, the lake bottom is covered with hydrophytes and the water quality is better than that in the algae-type zone [35].

Taihu Lake Basin has many important water source protection sites, providing drinking water for cities such as Suzhou City and Wuxi City. However, due to the rapid development of economy and population around Taihu Lake Basin, Taihu Lake has suffered increasing eutrophication in recent years. Since the outbreak of cyanobacteria in the water source of Gonghu Bay in 2007 [36], the government has taken a series of measures to rectify the Taihu Lake and achieved good results [37]. However, the frequency of cyanobacteria outbreak has not decreased [32], which seriously threatens the water supply security of the water source area.

### 2.2. Sampling Position

Samples were selected from the East Taihu Lake (Y1), Xukou Bay (Y2), Gonghu Bay (Y3, Y4 and Y5), Western shore-line (Y6). Y1–Y6 are important drinking water sources across the Taihu lake, serving 10 million people in the Taihu Lake Basin. Y1 (31°00.938′ N, 120°46.331′ E), Y2 (31°22.312′ N, 120°36.343′ E) and Y3 (31°38.505′ N, 120°39.054′ E) are located in grass-type zones with better water quality, surrounded by outflow rivers ; Y4 (31°45.001′ N, 120°37.775′ E), Y5 (31°40.434′ N, 120°24.028′ E) and Y6 (31°24.088′ N, 119°58.517′ E) are located in algal-type zones, especially in Y6, surrounded by main inflow rivers(Chengdonggang River, Guandugang River, etc.).

### 2.3. Sample Collection and Pretreatment

Surface sediment samples (0–10 cm) were collected near the drinking water intakes with a grab sampler in April 2015. Three adjacent surface sediment samples were collected from each site and mixed evenly in the field. These sediment samples were placed into sealed polyethylene tubes and temporarily stored in iceboxes at 4 °C. After immediate transfer to the laboratory, the samples were stored below −20 °C and then freeze-dried at −50 °C using FD-1D-50 freeze-dryers (Boyikang Corporation, Beijing, China). The dried samples were homogenized using an agate mortar and passed through a 100-mesh sieve before analysis.

The sediment samples were extracted using a 1 mol/L KCl solution (solid: water ratio of 1:10, *W*/*V*) for 1 h in a horizontal shaker at room temperature, then centrifuged at 5000 r/min for 15 min at 4 °C and filtered through 0.45-μm Millipore filters (mixed cellulose ester membrane) to remove suspended solids and residual bacteria. Extracts were stored in the dark, kept at 4 °C prior to the experiments, and analyzed within 1 weeks of collection

### 2.4. Sample Analysis

#### 2.4.1. KS-DON Analysis

NO_3_^−^-N was determined using ultraviolet (UV) spectrophotometry, whereas NH_4_^+^-N was measured using the salicylate hypochlorite method. TDN was measured via the alkaline potassium persulfate digestion-UV spectrophotometric method. All the determinations were done according to the Chinese National Standard Methods (SEPA of China., 2002) [38]. The concentration of KS-DON was calculated as the difference between the TDN and the sum of the inorganic nitrogen species (i.e., NO_3_^−^-N and NH_4_^+^-N) [39] (Equation (1)):

ω (KS-DON) = ω (TDN) − ω(NO_3_^−^-N) − ω (NH_4_^+^-N) (1)

where ω () is N concentration, N mg/kg. Each index was tested three times in parallel.

#### 2.4.2. Molecular Weight Fractionation

Extracts were fractionated using molecular sieves. Four types of regenerated cellulose membrane (Millipore Corp., Billerica, MA, USA) were used: (i) 1-kDa nominal molecular weight limit (NMWL); (ii) 3-kDa NMWL; (iii) 5-kDa NMWL; and (iv) 10-kDa NMWL. Extract samples at a volume of 200 mL were filtered through each membrane in series. Fifty milliliters of raw water and of each filtrate was retained for further analysis. The percentage of KS-DON in the samples collected using each membrane size was calculated as follows (Equations (2)–(6)):

(2) $$\%<1\;\mathrm{kDa}=\frac{C_{1k.permeate}}{C_{raw}}\times 100\left(\%\right)$$

(3) $$\%1k-3\;\mathrm{kDa}=\frac{C_{3k.permeate-}C_{1k.permeate}}{C_{raw}}\times 100\left(\%\right)$$

(4) $$\%3k-5\;\mathrm{kDa}=\frac{C_{5k.permeate-}C_{3k.permeate}}{C_{raw}}\times 100\left(\%\right)$$

(5) $$\%5k-10\;\mathrm{kDa}=\frac{C_{10k.permeate-}C_{5k.permeate}}{C_{raw}}\times 100\left(\%\right)$$

(6) $$\%>10\;\mathrm{kDa}=\frac{C_{raw-}C_{10k.permeate}}{C_{raw}}\times 100\left(\%\right)$$

where “*C*” represents the concentration of KS-DON under each molecular weight.

Based on Yeh et al.’s [40] study, we divided the sediment DON into two groups, namely high molecular weight KS-DON (HMW, MWs >1 kDa) and low molecular weight KS-DON (LMW, MWs <1 kDa).

#### 2.4.3. Hydrophilic and Hydrophobic Fractionation

**Figure 2:**
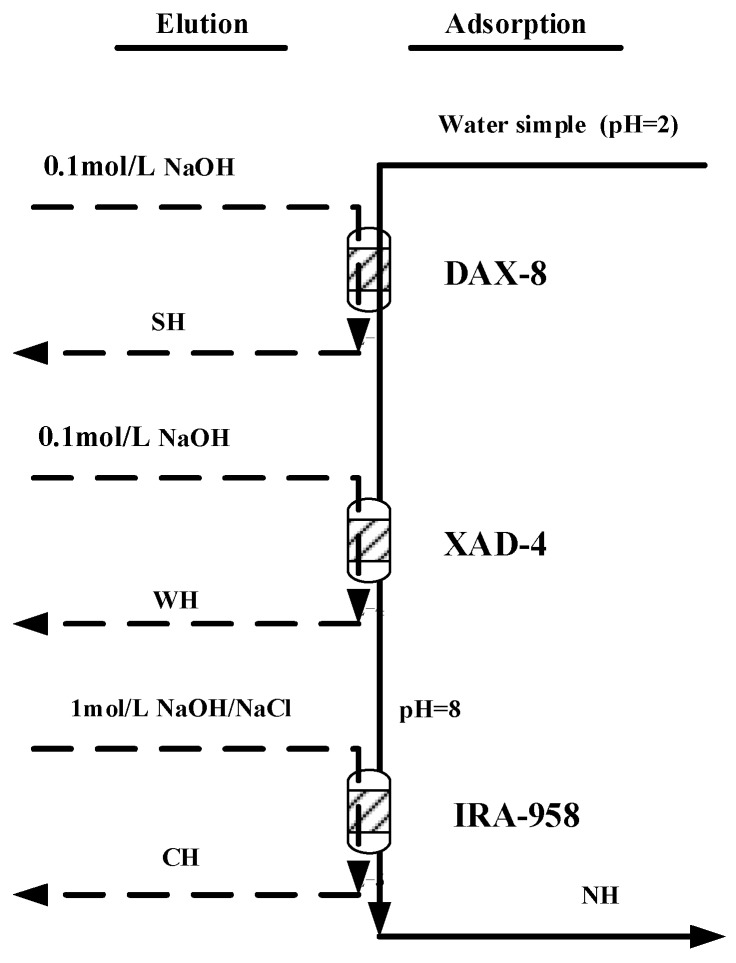
Outline of the water sample hydrophobicity fractionation procedure.

Extract was fractionated by using different resins (Supelite DAX-8, Amberlite XAD-4 and Amberlite IRA-958, Sigma-Aldrich, St. Louis, MO, USA) [41,42]. The fractionation approach suggested by Tao et al. [16] was used and is shown schematically in Figure 2. Each extracts was adjusted to pH = 2 and fed onto a Supelite DAX-8 non-functionalized resin, which retained strongly hydrophobic (SH) organic matter. This fraction was eluted with 0.1 mol/L NaOH. The unabsorbed concentrate from the DAX-8 resin was fed onto an Amberlite XAD-4 resin, which retained weakly hydrophobic (WH) organic matter. This fraction was also eluted with 0.1 mol/L NaOH. The unabsorbed concentrate from the XAD-4 resin, which comprised hydrophilic organic matter attributed to proteins, amino acids, and carbohydrates, was fed onto an Amberlite IRA-958 anion exchange resin, which retained charged hydrophilic (CH) material. This fraction was eluted with 1 mol/L NaOH/NaCl. The remaining neutral hydrophilic (NH) material was not retained by any of the resins.

#### 2.4.4. EEM Fluorescence Spectroscopy Determination and Analysis

Three-dimensional excitation-emission matrix (EEM) fluorescence spectroscopy (F-700 FL spectrophotometer, Hitachi, Tokyo, Japan) was used to characterize KCl-extractable sediment DOM. The excitation (Ex) wavelength was set from 200 to 450 nm at 5-nm intervals, corresponding to emission (Em) wavelengths from 250 to 550 nm at the same intervals. The scanning speed was set at 1200 nm/min. The spectrum of deionized water was recorded as the blank.

Fluorescence regional integration (FRI), a quantitative technique proposed by Chen et al. [43], integrates volumes beneath different Ex-Em regions in EEMs, and can be used to quantitatively analyze EEMs and determine the configuration and heterogeneity of DOM. The EEM spectrum was divided into five regions (Table 1), including simple aromatic proteins, such as tyrosine and tryptophan (Regions I and II), fulvic acid-like substances (Region III), related to soluble microbial byproduct-like materials (Region IV), and humic acid-like organics (Region V).

### 2.5. Data Analysis

The water quality parameters of extracts were expressed as the mean ± standard deviation of the three replicate samples. One-way analysis of variance (ANOVA) was used to analyze the differences of nutrient in the sediment between six sites. Statistical analysis was performed using the SPSS 20.0 statistical package (IBM corp., Armonk, NY, USA), and the level of significance used was *p* < 0.05 for all tests.

## 3. Results and Discussion

### 3.1. Sediment Nitrogen Species and Concentrations

**Figure 3:**
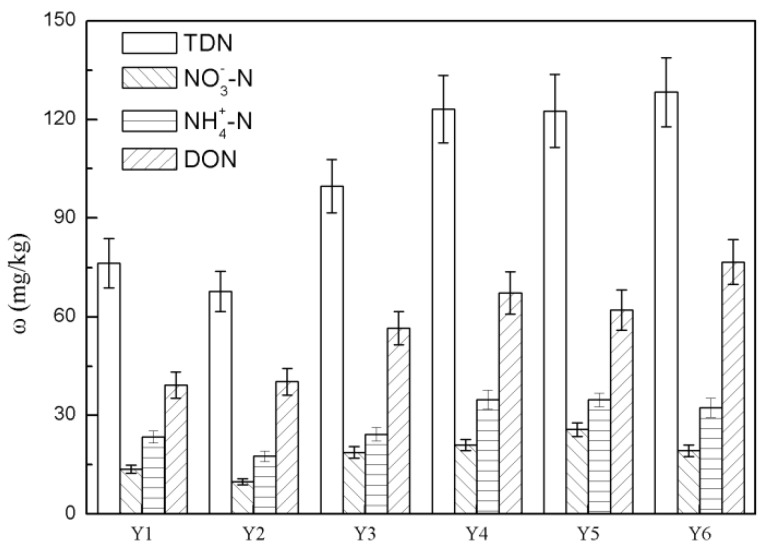
The distribution and forms of nitrogen in each sediment sample.

The nitrogen species and concentrations in each extracts are shown in Figure 3. The concentrations of NO_3_^−^-N showed no significant differences (*p* > 0.05) between six sites, while the concentrations of TDN, NH_4_^+^-N and DON differed (*p* < 0.05), and the TDN, NH_4_^+^-N and DON content of sites in the Algae-type zones were higher (*p* < 0.05) than the Grass-type zone. The content of TDN showed as follows: Y6 > Y5 > Y4 > Y3 > Y2 > Y1. The concentrations of TDN in the six sediment samples from Taihu Lake ranged from 67.78 mg/kg to 128.27 mg/kg. The sediment NH_4_^+^-N content ranged from 17.64 mg/kg to 34.78 mg/kg, and accounted for 24–31% of sediment TDN content. Its distribution pattern across the six sample sites was the same as that of sediment TDN. The sediment NO_3_^−^-N content ranged from 9.87 mg/kg to 25.70 mg/kg, and accounted for the lowest percentage fraction of sediment TDN compared with other nitrogen species. It is likely that this result is because the sediment is an anaerobic environment, which is unfavorable to the formation of NO^3−^-N sediment NO^3−^-N. The KS-DON content ranged from 39.23 mg/kg to 76.63 mg/kg, contributed to 68.0–82.5% of sediment TDN, consistent with previous studies [14].

The sediment DON content of the Taihu Lake is closely related to the level of pollution, and research has shown that the more serious the pollution, the higher the concentration of DON in the sediment [6]. Sediment release is an important source of DON in surface water, which can be directly absorbed by algae, leading to eutrophication, and even algae blooms [44].

### 3.2. Molecular Weight Fractionations of KS-DON

**Figure 4:**
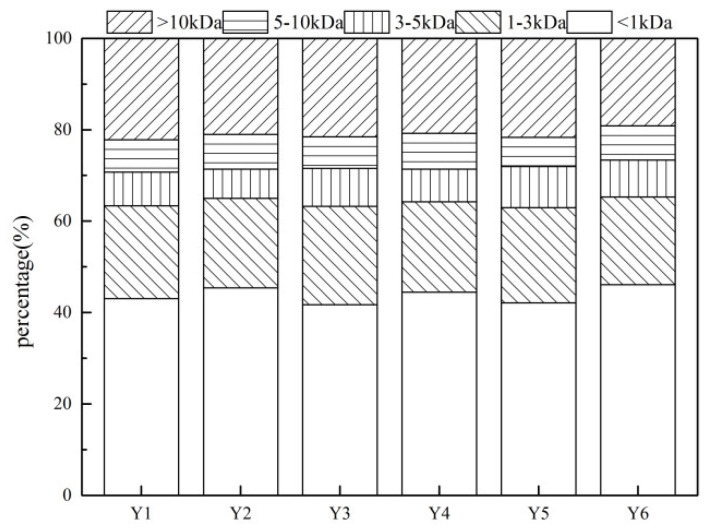
Molecular weight fractionations of KS-DON.

The KS-DON composition of each molecular weight fraction is shown in Figure 4. Although the KS-DON concentration across the six sample sites varied, there was no obvious difference in the molecular weight distributions of KS-DON. The molecular weight fractions of <1 kDa accounted for almost half of KS-DON (43.78 ± 1.51%). The molecular weight fractions of 1–3 kDa and >10 kDa were considerable, with mean values of 20.22 ± 0.67% and 21.06 ± 0.74%, respectively. The fractions of 3–5 kDa and 5–10 kDa were relatively small, accounting for 7.71 ± 0.75% and 7.23 ± 0.42% of KS-DON, respectively, which is different with Erhai Lake. The fractions of <1 kDa accounted for only 12.3% in the Erhai Lake sediment [12], which is high molecular weight fraction accounted majority.

The results showed that KS-DON of the Taihu Lake mainly comprised small molecular weight fractions, which is similar to the results of Tao et al. [16] and showed that most of the DON released from sediment comprised fractions <3 kDa, accounting for approximately 77.5% of the total DON [16]. Small-molecule DON, including proteins, amino acids, nitro and heterocyclic compounds, can easily be released into raw water, increasing the difficulty of removing DON using current water treatment technologies.

### 3.3. Hydrophilic and Hydrophobic Fractions of KS-DON

**Figure 5:**
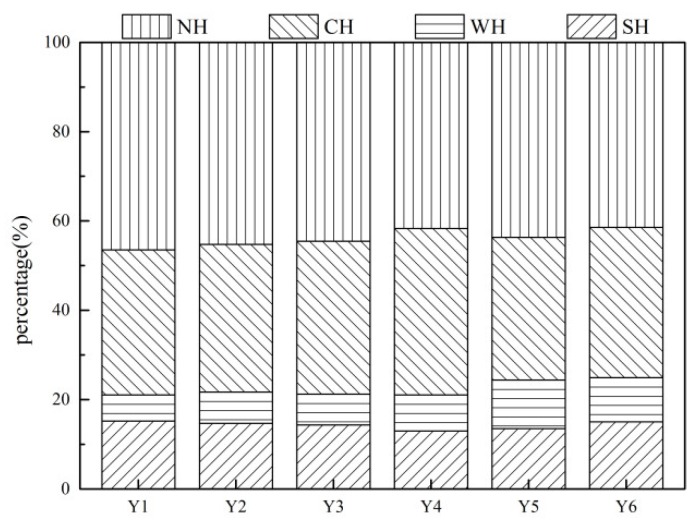
Hydrophilic and hydrophobic fractions of KS-DON.

According to the published literature, humic and fulvic acids are the main components of SH, and hydrophilic (non-humic) organic matter (CH and NH) are attributed to proteins, amino acids, and carbohydrates [45,46]. As shown in Figure 5, the distribution of hydrophilic and hydrophobic fractions of KS-DON was similar across samples. The NH fraction was the main component, contributing 43.84 ± 1.57% of KS-DON in all sample sites. CH was the second main component of KS-DON, contributing 33.78 ± 1.32%, followed by SH and WH (14.29 ± 0.69% and 8.10 ± 1.51%, respectively). Previous study also indicated that approximately 62.74% of the KS-DON was hydrophilic fraction in Taihu Lake [14].

The KS-DON fractionation results demonstrated that KS-DON from Taihu Lake mainly comprised hydrophilic groups. Research has shown that plant and animal debris can degrade to proteins, polypeptides, and amino acids, increasing the content of hydrophilic DON [16]. The hydrophilic portion can be more easily released to water compared with the hydrophobic portion, thus increasing the concentration of DON in water and making water treatment more difficult [47].

### 3.4. Fluorescence Characteristics of KS-DON

**Figure 6:**
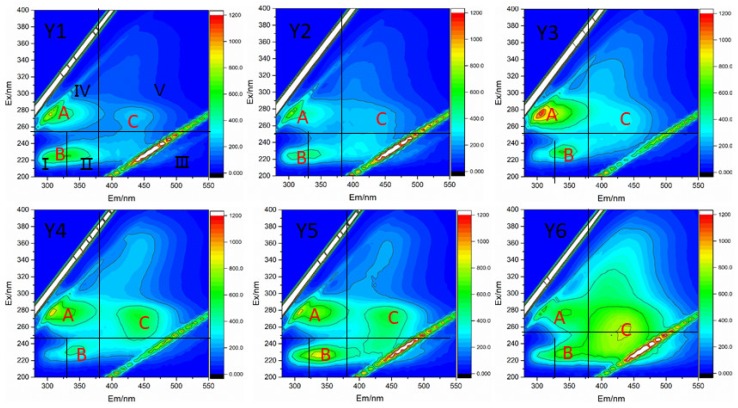
3D fluorescence maps of DON from each sample site.

Figure 6 gives the 3D fluorescence spectra of KS-DON in the six sediment samples from Taihu Lake. From the EEM spectra, three main peaks were identified: (i) peak A was observed at Ex/Em wavelengths of 275–285/310–320 nm, representing soluble microbial by products produced by the fluorescence of microbial metabolites, including proteins, coenzymes, low-molecular-weight organic acids, and pigments [43,48]; (ii) peak B was observed at 220–230/330–340 nm, which is the boundary of Region I and II and represents tyrosine and tryptophan-like proteins. Peak B also represents protein-like substances formed by endogenous products mainly resulting from the degradation of enzymes or biological debris [49]. Tyrosine-like proteins are mainly related to aromatic protein-like structures generated from microbial degradation, whereas tryptophan-like proteins are mainly related to aromatic amino acid structures and degradation products of lignin [50]; and (iii) Peak C was observed at Ex/Em values of 270–280/420–430 nm, which was thought to be generated mainly by humic acid-like substances with complex molecular structures [51], this substance is usually considered as nondegradable fractions for its complex and stable structure with aromatic ring and covalent bond. The result was similiar to Su et al.’s [52] research results on EEMs of KS-DON in Shankou Lake, which two types of protein-like substances and one FA-like substance were observed at Ex/Em values of 225–275/350, 275/240–350 and 250/400–410.

The distributions of volumetric fluorescence among the five regions (P_i,n_) for the KS-DON are presented in Table 2. Fluorescence components of KS-DON from water sources (except Y6) of Taihu Lake mainly comprised simple aromatic proteins (Region I and II) with fewer humic acid-like substances (Region V) and fluvic acid-like substances (Region III), reflected by its high ratio of P_I+II_,n for region of simple aromatic proteins(region I and II), ranging from 50.13% to 61.76%. This quantitative result was consistent with visual analysis of the location of peaks within regions I and II of the EEM spectra (Figure 6). While fluorescence components of KS-DON from Y6 mainly comprised fluvic acid-like substances (Region III) and humic acid-like substances (Region V), which was different with other water source KS-DON. Previous studies reported that main fluorescence peak of hydrophilic fraction in Taihu Lake surface sediment extracts were located at Region II and III, whereas hydrophilic fraction has another obvious fluorescence peak in Region IV [14]. Besides, there was a obvious fluorescence peak on Region IV of LMW fractions, whereas a humic-like substance peak was obvious in the Region V of HMW fractions [12].

Here, the ratio of P_III+V,n_ for the humic- and fulvic-like regions (regions III and V) to P_I+II+IV,n_ for the protein-like regions (regions I, II and IV) were calculated (Table 2),ranging from 0.32 to 0.8,which is much lower than the ratio of the Erhai Lake sediment(ranging from 1.30 to 2.56) [12]. This indicated the higher humid material proportion and the correspondent more stable property in Erhai sediment than in Taihu Lake. Qian et al. [53] also reported that the structural groups of organic nitrogen in Taihu Lake sediments were relatively complex, but the aromaticity was low and the aromatic ring substituents were few. Therefore, the retention capacity of nutrients was weak in Taihu Lake sediments, which contributes greatly to water pollution.

In addition, although the sediments of Y1, Y2 and Y3 had lower P_III+V,n_/P_I+II+III,n_ and higher risk of DON endogenous release, the water quality of Y1, Y2 and Y3 was significantly better than that of other water sources, Wan et al. [54] have reported that the annual variation of total nitrogen concentration in Western Taihu Lake ranged from 2.25 mg/L to 3.41 mg/L, but for Eastern Taihu Lake and Xukou bay, the annual variation range of total nitrogen concentration is only between 0.25 mg/L and 1.34 mg/L. The difference of Lake type is one of the important reasons for this difference [34], because of the flourishing of aquatic plants and the absorption of nutrient release in the Grass-type zones [54], which is important for reduction of nitrogen release and protection of water sources.

### 3.5. Possible Management Approaches on Reduction the Risk of Endogenous N Release

From the above, we concluded that KS-DON in Taihu Lake source sediments is mainly composed of hydrophilic and small molecular components, while the fluorescence spectrum of KS-DON shows high protein fluorescence. Based on the analysis of Zhai et al. [12,14,52,55], it is concluded that the sediment KS-DON in Taihu Lake source area has higher bioavailability and higher risk of endogenous release. In addition, higher NH_4_^+^-N loads also exist in sediments, which should be paid attention to. In view of this, we propose the following possible measures to reduce the release of endogenous nitrogen.

#### 3.5.1. Ecological dredging

The purpose of ecological dredging is to remove surface sediments containing high-impact salts, including suspended, semi-suspended flocculent colloids of nutrients deposited on the surface of silt, or dormant algae or biological debris of animals and plants. Liu et al. [56] have reported that ecological dredging would effectively control N-release in Wuli Lake after interception the external loading. Liu et al.’s [57] research also confirmed the role of ecological dredging in reduction of organic matter content in sediments and weakening total biological activity, but it’s worth noting that there was a high risk of endogenous NH_4_^+^-N release (especially at higher water temperature) in the short term after ecological dredging, because of the higher NH_4_^+^-N content in the porewater, so it was suggested that ecological dredging in winter with lower water temperature could reduce the internal loading in order to achieve positive efficiency.

#### 3.5.2. Establishment of constructed wetlands

Wetlands and higher aquatic plants can play the role of wave dissipation and physical retardation, thus promoting particulate deposition and reducing sediment resuspension [58]. Besides, aquatic plants can absorb a lot of nutrients in water and sediments, and some macrophytes could significantly improve the removal efficiency benthic N by enhancing the ANAMMOX and denitrification processes [27]. Moreover, constructed wetlands have the value of resource utilization and can effectively improve the ecological environment around Taihu Lake.

## 4. Conclusions

The characteristics of KS-DON in the six drinking water sources from Taihu Lake were investigated in the current study. KS-DON was the main species of TDN and accounted for >50% of TDN. Molecular weight fractions of <1 kDa, 1–3 kDa, 3–5 kDa, 5–10 kDa, and >10 kDa accounted for 43.78%, 20.22%, 7.71%, 7.23% and 21.06% of TDN, respectively. NH was the main component of KS-DON and averagely contributed 43.84% of KSDON in the sediment samples. CH, SH, and WH contributed 33.78%, 14.29%, and 8.09% of KS-DON, respectively. There were three fluorescence peaks: (i) soluble microbial byproducts; (ii) protein-like substances; (iii) and humic acid-like substances. It is concluded that KS-DON in Taihu Lake is mainly hydrophilic and low molecules with high bioavailability, low humification and high risk of KS-DON endogenous release. Therefore, measures should be taken not only to control the input of exogenous pollution, but also to reduce the risk of endogenous N-pollution release, such as using ecological dredging and establishment of constructed wetlands.

## Figures and Tables

**Table 1:** Excitation and emission (Ex/Em) wavelengths for each fluorescence region.

Region	Substance	Ex/EM Wavelengths (nm)
I	Tyrosine-like proteins	200–250/280–330
II	Tryptophan-like proteins	200–250/330–380
III	Fulvic acid-like organics	200–250/380–550
IV	Soluble microbial byproducts	250–400/280–380
V	Humic acid-like organics	250–400/380–550

**Table 2:** Percentage distribution of (f_i_ × 100%) of KS-DON.

	P_I,n_	P_II,n_	P_III,n_	P_IV,n_	P_V,n_	P_III + V,n_/P_I +II + III,n_
Y1	28.46	33.34	13.71	13.99	10.51	0.32
Y2	25.85	31.51	17.24	15.67	9.74	0.37
Y3	19.44	30.69	11.58	21.64	16.65	0.39
Y4	13.24	40.65	12.65	15.32	18.15	0.44
Y5	21.07	33.33	16.00	14.37	15.23	0.45
Y6	13.72	29.76	23.09	11.95	21.48	0.80
